# Supplementary material for: Effect of senior executives’ overseas experience on corporate green innovation
Source: PLoS One. 2024 May 2;19(5):e0296716. doi: 10.1371/journal.pone.0296716 (PMC11065230; doi:10.1371/journal.pone.0296716)
Supplement: Attachment — Submitted filename: Reviewer.docx [file pone.0296716.s002.docx]

**Referee report**

Manuscript title: **Effect of Senior Executives’ Overseas Experience on Corporate Green Innovation**

**Summary**

The paper studies how senior executives’ oversea experience influences corporate green innovation. By collecting yearly data of listed A-share companies in China from 2007 to 2019, the data sample contains 15,389 panel data of 2,681 companies. The results showed that senior executives’ overseas experience could promote green innovation in companies. This positive effect was more significant for private enterprises and high-tech enterprises, especially in Eastern China. This study enriches upper echelons theory and provides theoretical support for government agencies to accelerate innovative green development strategies

**General comments:**

- The topic is nice and worthwhile studying. The contribution that this paper aims to present is also noticeable when examining the impact of oversea experience of senior executives on the corporate green innovation.
- The time chosen for the data sample is from 2007 to 2019. The author(s) might have intendedly neglected the potential bias from the global financial crisis (2008/2009). Thus, I would consider that the author(s) should consider the impact of the global financial crisis on data analysis.

**Major comments:**

- The research background and research motivation are not clear. For example, many studies (???) have investigated the effect of executives’ personal characteristics and background on corporate innovation.
- For literature review, the author(s) have not introduced background of corporate green innovation and theories related to the relationship between executive’s overseas experience and corporate green innovation. Even though the study mentioned upper echelons theory, the author(s) can think of applying more theories like imprinting theory following previous studies (He, Chen & Zhang, 2021; Quan, Quian & Zhang, 2021).
- I think that the paper may need check data sample. The author(s) used a sample of 15,389 observations from 2,681 companies. However, according to the descriptive statistics, the highest observations of variables is less than 15,389. The author(s) should double check the data sample.
- I am just wondering how the author(s) define executives? Do senior executives include chairman, CEO, COO…? The author(s) divided executives to sub-sample including senior managers and junior managers. I suppose that chairman and/or CEO’s oversea experience have strong impact on corporate green innovation. The author(s) can think of investigating the moderating role of chairman and CEO. Furthermore, the author(s) should think of the title: senior executives or executives or senior managers or managers.
- The author(s) used panel data. However, the model did not show the panel data. Furthermore, fixed effect model (FEM) is a statistic are technique to analyse data. The author(s) should explain why the author(s) use FEM to analyse data rather than adding fixed effects to the research model.
- The author(s) described the results well. However, the discussion is not clearly. The author(s) should focus on how the findings link with theories and previous studies.
- The author(s) did many robust tests. However, the author(s) should use sub-sample to explain the reasons for using these robust tests.

**Minor points:**

- The author(s) may need to introduce correlation matrix.
- The author(s) can consider GMM/2SLS for endogeneity.

**Overall:** The paper is nice. The topic is fascinating. Although there are always spaces of paper improvement, this paper needs to seriously consider these matters. Good luck!

**References**

He, K., Chen, W., & Zhang, L. (2021). Senior management's academic experience and corporate green innovation. *Technological Forecasting and Social Change*, *166*, 120664.

Quan, X., Ke, Y., Qian, Y., & Zhang, Y. (2021). CEO foreign experience and green innovation: evidence from China. *Journal of Business Ethics*, 1-23.
