# Supplementary material for: Effect of senior executives’ overseas experience on corporate green innovation
Source: PLoS One. 2024 May 2;19(5):e0296716. doi: 10.1371/journal.pone.0296716 (PMC11065230; doi:10.1371/journal.pone.0296716)
Supplement: Attachment — Submitted filename: Response to Reviewers.docx [file pone.0296716.s003.docx]

****Response to reviewer** comments**

Dear Reviewer,

We are very grateful for your favorable view on our manuscript. Your comments are very important to the acceptability of our paper. According to your suggestions, we have made the following revisions on this manuscript:

**General comments:**

- The topic is nice and worthwhile studying. The contribution that this paper aims to present is also noticeable when examining the impact of oversea experience of senior executives on the corporate green innovation.
- The time chosen for the data sample is from 2007 to 2019. The author(s) might have intendedly neglected the potential bias from the global financial crisis (2008/2009). Thus, I would consider that the author(s) should consider the impact of the global financial crisis on data analysis.

**Response：**

**Indeed, there is a lack of summary of our contributions to the paper. Based on your suggestion, we have added a description of our contributions at the end of the introduction.**

**In order to avoid potential biases caused by the global financial crisis, we controlled for the impact of this factor in the robustness testing section. The specific content is as follows:**

**Major comments:**

- The research background and research motivation are not clear. For example, many studies (???) have investigated the effect of executives’ personal characteristics and background on corporate innovation.

**Response：**

**Our background and motivation are mainly based on green and innovation, and currently the academic community mostly studies the two separately, without analyzing them in a unified structure. We have added an explanation to this in the second paragraph of the introduction.**

- For literature review, the author(s) have not introduced background of corporate green innovation and theories related to the relationship between executive’s overseas experience and corporate green innovation. Even though the study mentioned upper echelons theory, the author(s) can think of applying more theories like imprinting theory following previous studies (He, Chen & Zhang, 2021; Quan, Quian & Zhang, 2021).

**Response：**

**We have added existing research on imprinting theory in the literature review section.**

- I think that the paper may need check data sample. The author(s) used a sample of 15,389 observations from 2,681 companies. However, according to the descriptive statistics, the highest observations of variables is less than 15,389. The author(s) should double check the data sample.

**Response：**

**We have checked the data sample and corrected the observations. Now, our sample observations is 15334. It should be noted that some variables have missing observations, so not all variables have observations of 15334.**

- I am just wondering how the author(s) define executives? Do senior executives include chairman, CEO, COO…? The author(s) divided executives to sub-sample including senior managers and junior managers. I suppose that chairman and/or CEO’s oversea experience have strong impact on corporate green innovation. The author(s) can think of investigating the moderating role of chairman and CEO. Furthermore, the author(s) should think of the title: senior executives or executives or senior managers or managers.

**Response：**

**We define executives according to China's Company Law, which includes the chairman, CEO, and COO. Indeed, our empirical evidence also indicates that the impact of senior managers is significant and more important as you mentioned.**

**Examining the moderating effect of senior managers is indeed a good suggestion. Unfortunately, multicollinearity may be encountered when conducting regulatory effect analysis. The overseas experiences of executives themselves are divided into two categories: senior executives and junior executives, and the interaction term may be omitted in regression process, so we did not conduct this analysis. However, don't worry, our results have shown that senior executives are more influenced.**

- The author(s) used panel data. However, the model did not show the panel data. Furthermore, fixed effect model (FEM) is a statistic are technique to analyse data. The author(s) should explain why the author(s) use FEM to analyse data rather than adding fixed effects to the research model.

**Response：**

**We have edited the model again, as shown in Eq (1).**

**In the robustness testing section, we also added explanations on why FEM is used.**

- The author(s) described the results well. However, the discussion is not clearly. The author(s) should focus on how the findings link with theories and previous studies.

**Response：**

**We have added a connection with previous theories and studies in the explanation of the results.**

- The author(s) did many robust tests. However, the author(s) should use sub-sample to explain the reasons for using these robust tests.

**Response：**

**We have used sub-sample to explain the reasons for using these robust tests. The specific content is as follows:**

**Overall, the overseas experience of executives has indeed enhanced the green innovation of the enterprise. However, the sub-sample analysis showed significant heterogeneity, does this make our results unreliable? We need to conduct a series of robustness tests to prove the reliability.**

**Minor points:**

- The author(s) may need to introduce correlation matrix.

**Response：**

**We have added a correlation matrix.**

- The author(s) can consider GMM/2SLS for endogeneity.

**Response：**

**We have selected instrumental variables in the robustness testing section and conducted 2SLS analysis. This greatly solves endogeneity.**
