# Supplementary material for: Effect of senior executives’ overseas experience on corporate green innovation
Source: PLoS One. 2024 May 2;19(5):e0296716. doi: 10.1371/journal.pone.0296716 (PMC11065230; doi:10.1371/journal.pone.0296716)
Supplement: Attachment — Submitted filename: Response to Reviewers.docx [file pone.0296716.s005.docx]

****Response to reviewer #1** comments**

Dear Reviewer #1,

We are very grateful for your favorable view on our manuscript. Your comments are very important to the acceptability of our paper. According to your suggestions, we have made the following revisions on this manuscript:

1. Introduction section is still immature. You need to clearly layout the introduction section in a way that it clearly summarizes what you did in this study, what is the research gap, what you found, then the contribution, and the brief structure of the paper. Please follow the following great paper by Professor Bellemare to restructure and develop your Introduction section more. I am sure it will benefit you.
http://marcfbellemare.com/wordpress/wp-content/uploads/2020/09/BellemareHowToPaperSeptember2020.pdf

**Response 1：**

**We have carefully read Professor Bellemare's paper, and based on your suggestions and Professor Bellemare's views, we have edited the introduction section again.**

2. Explanation of the reason why choosing the IV: How the chosen IV meet the exclusion restrictions of IV/2SLS estimation? If not explaining it carefully, the IV is not valid. Moreover, the author(s) must provide the statistics of the under-identification and weak identification tests for the IV-2SLS estimation. It is mandatory when use IV/2SLS regression.

**Response 2：**

**We have added relevant explanations in the IV-2SLS section and added the statistics of the under-identification and weak identification tests.**

3. Numbering sections: The current section and sub-section structure is hard to follow. Please number them consecutively.

**Response 3：**

**We have reconfirmed the coherence between section and subsection.**

4. Presentation of the paper: I see numerous grammar and typos in the manuscript. For examples:
- Data section: Data collected from "Cathay Pacific"? I think you meant CSMAR?
- "Table10", "Table11" with no space between "Table" and the table number.

**Response 4：**

**We carefully checked and corrected grammar and typos.**

****Response to reviewer #3** comments**

Dear Reviewer #3,

We really appreciate you for your carefulness and conscientiousness. Your suggestions are really valuable and helpful for revising and improving our paper. According to your suggestions, we have made the following revisions on this manuscript:

1. The author/s/ of the paper seem to have made significant improvements based on the feedback received during the first round of revisions. However, upon conducting a meticulous examination of the paper, it becomes apparent that the study primarily focuses on the relationship between overseas executives and green innovation (GI), a topic that has already been extensively discussed and debated in the existing literature. This review points out the need for further refinement and expansion of the paper's content, particularly in the area of exploring the factors that drive green innovation beyond the involvement of foreign executives.
One notable observation is that the paper concludes that green innovation can be driven by the presence of foreign executives (GI could be driven by having foreign executive GI). While this conclusion has merit, it's crucial to consider the broader context and the numerous variables that influence green innovation. There's a rich body of literature that links green innovation to CEO and executive actions, specifically related to CEO pay regulation. Surprisingly, the author(s) have omitted this critical aspect from their hypotheses and subsequent analysis.
To address this omission effectively, the author(s) should include a subsection dedicated to the drivers of green innovation within the paper. This subsection should delve into the research on the influence of CEOs and executives, as well as the role of CEO pay regulation in promoting or hindering green innovation initiatives. By incorporating this aspect, the paper would be more comprehensive and aligned with the current state of research in this field. To further enhance the study's comprehensiveness, the author should consider including the following relevant studies:
• Li, Q., Maqsood, U. S., & Zahid, R. M. A. (2023). "Nexus between government surveillance on executive compensation and green innovation: Evidence from the type of state-owned enterprises." Published in *Business Ethics, the Environment & Responsibility*, this study provides insights into the relationship between government oversight of executive compensation and green innovation.
• Li, Q., Maqsood, U.S., Zahid, R.A., et al. "Regulating CEO pay and green innovation: moderating role of social capital and government subsidy" (2023). Published in the *Environmental Science and Pollution Research*, this study explores the regulatory aspects of CEO pay and its impact on green innovation.
These recent studies are directly related to the topic at hand and have formulated their hypotheses accordingly.

**Response 1：**

**We have added a section that references the research of Li et al. (2023a,2023b), specifically designed to examine the moderating effect of CEO pay regulation. The specific content is as follows:**

2. One major concern raised by the reviewer in R1 is regarding the IV-2SLS. The author claims to have used the term "clone," but it is unclear how this term is defined and how it relates to the current scenario in the context of green innovation? I strongly recommend that the author addresses these points seriously and revises the manuscript with a more comprehensive exploration of incentives and the suggested improvements to strengthen the narrative.

**Response 2：**

**We have added relevant explanations in the IV-2SLS section. Furthermore, we have strengthened the narrative of the manuscript.**

**References:**

Li Q, Maqsood US, Zahid RMA. Nexus between government surveillance on executive compensation and green innovation: Evidence from the type of state-owned enterprises.*Business Ethics, the Environment & Responsibility*. 2023a. https://doi.org/10.1111/beer.12601

Li Q, Maqsood US, Zahid RMA, Anwar W.Regulating CEO pay and green innovation: moderating role of social capital and government subsidy. *Environmental Science and Pollution Research*. 2023b. https://doi.org/10.1007/s11356-023-26641-x
